# Technological and Biological Reliability, and Validity of Five Different CPET Systems During Simulated and Human Exercise

**DOI:** 10.1111/sms.70184

**Published:** 2026-01-24

**Authors:** Bas Van Hooren, Tjeu Souren, Félix Miqueu, Bart C. Bongers

**Affiliations:** ^1^ Department of Nutrition and Movement Sciences, Institute of Nutrition and Translational Research in Metabolism (NUTRIM) Maastricht University Maastricht the Netherlands; ^2^ Independent Consultant Utrecht the Netherlands; ^3^ Department of Surgery, Institute of Nutrition and Translational Research in Metabolism (NUTRIM) Maastricht University Maastricht the Netherlands

**Keywords:** graded exercise testing, metabolic cart, precision, reliability, simulation, validity

## Abstract

The validity and between‐day reliability of cardiopulmonary exercise testing (CPET) systems remain largely unexplored. We therefore evaluate the validity and between‐day technological and biological reliability of five popular CPET systems for assessing respiratory variables, substrate use, and energy expenditure during simulated and real human exercise. The following systems were assessed: Vyntus CPX, Oxycon Pro, VO2 Master, KORR, and Calibre. A metabolic simulator was used to simulate breath‐by‐breath gas exchange. The values measured by each system (minute ventilation (V̇E), breathing frequency (BF), oxygen uptake (V̇O_2_), carbon dioxide production (V̇CO_2_), respiratory exchange ratio (RER), energy from carbohydrates and fats, and total energy expenditure) were compared to the simulated values to assess the validity. Six well‐trained participants cycled 5% below their first ventilatory threshold on 2 days to verify the validity in human exercise. Between‐session reliability was assessed in both the simulation and human experiments to determine technological and biological variability. Absolute percentage errors during the simulations ranged from 0.69% to 5.56% for V̇E, 0.92% to 1.44% for BF, 3.12% to 7.86% for V̇O_2_, 4.07% to 12.1% for V̇CO_2_, 1.21% to 6.94% for RER, 2.83% to 48.8% for Kcal from carbohydrates, 14.1% to 50.3% for Kcal from fats, and 4.21% to 6.98% for total energy expenditure. Between‐session variability during simulation (i.e., technological variability) ranged from 0.46% to 3.15% for V̇O_2_ and 0.71% to 4.99% for V̇CO_2_. The error and between‐day variability of the error for respiratory gas variables, substrate, and energy use differed substantially between systems. Biological and technological V̇O_2_ and V̇CO_2_ variability, respectively, accounted for ~60%–70% and 40%–30% of the variability in repeated human testing.

## 1 Introduction

Cardiopulmonary exercise testing (CPET) is an important tool for evaluating cardiorespiratory fitness and tolerance to progressive exercise up to volitional exhaustion in both clinical [1, 2, 3, 4, 5] and athletic populations [6, 7]. By measuring variables such as oxygen uptake (V̇O_2_), carbon dioxide production (V̇CO_2_), and minute ventilation (V̇E), CPET can be used to assess physiological variables and indices, like the first and second ventilatory thresholds [8, 9, 10], maximal oxygen uptake (V̇O_2max_) [7, 11, 12], oxygen uptake kinetics [13], substrate utilization [14, 15], and total energy expenditure [16] during exercise. For example, clinicians use CPET to diagnose cardiovascular and respiratory conditions, or to assess the risk of strenuous treatments (e.g., surgery, chemotherapy) [1, 2, 17], while athletes and coaches use CPET to set training intensities and evaluate performance. Additionally, CPET is often regarded as the gold‐standard reference method for validating other methods that estimate cardiorespiratory fitness.

Many CPET systems are available on the market, each using different technologies to measure flow or volume, or to measure O_2_ and CO_2_ concentrations, and algorithms to process data from these sensors, which can affect accuracy and reliability [18, 19, 20, 21, 22]. Variation in technologies and algorithms, along with the resulting differences in accuracy and reliability, means each system needs to be evaluated to assess its performance under different conditions.

The accuracy and reliability of CPET systems can be tested through combustion tests [23, 24, 25], metabolic simulators [18, 26, 27, 28, 29, 30, 31, 32, 33, 34], and direct comparisons in human testing [18, 24, 35, 36, 37, 38] (including comparisons to Douglas bag testing [39, 40, 41, 42]), with each method having its own strengths and weaknesses [18]. Among these methods, metabolic simulators and direct comparisons during human exercise testing are considered more relevant to real‐world applications than combustion tests [18]. Accordingly, several studies have combined metabolic simulations with human exercise testing to assess the accuracy and/or reliability of different CPET systems [18, 33, 43, 44]. One recent study [18], for example, examined the accuracy—and in some cases, the reliability—of 15 different CPET devices, providing the most comprehensive overview of products available in the current CPET market to date. Of particular interest in this study is that one of the systems tested (Calibre) showed high accuracy in both simulation and human exercise comparisons. Because this system is also wearable and affordable (~465 euro), it has gained significant popularity among practitioners and researchers. However, its actual performance in daily practice has received mixed feedback from physiologists, clinicians, scientists, and coaches, with various users reporting implausible data during apparently well‐controlled exercise testing (personal communication with the present study authors). The discrepancy between study findings and field results observed for this device may reflect users errors, but could also be due to modifications in the algorithms and technology that have occurred since the original research was conducted and indicate the need for another evaluation of the device's accuracy. Conversely, the VO2 Master Pro device demonstrated relatively poor accuracy in that study [18], which contrasts with the findings of a recent study showing high accuracy and reliability [44]. While this better performance may reflect updates to the algorithms (e.g., a metabolic simulator mode enabling direct comparison with a metabolic simulator), this study also corrected for differences in measured humidity, temperature, and pressure between the VO2 Master Pro and the metabolic simulator, which can artificially increase accuracy. Indeed, such differences are inherent to the CPET system and should not be replaced with data from the metabolic simulator, because the humidity, temperature, and pressure measured by the CPET device are also used to derive correction factors during actual human testing to convert the measured V̇O_2_ and V̇CO_2_ from expired breath conditions to STPD. Consequently, an updated evaluation for this device without correction of such differences is warranted. Therefore, a new evaluation of the accuracy and reliability of these devices is needed, along with an assessment of devices not included in the previous study. The present study, therefore, aimed to evaluate the accuracy of five different CPET devices under metabolic simulation and to verify these results during human cycling tests.

For repeated exercise tests, high reliability may be more important than high accuracy. The reliability of many CPET systems remains however unknown, or it has been investigated using repeated human tests where biological variability might obscure the true variability of the system. Katch and co‐workers [45], for example, reported a total between‐day variability of 5.6% for V̇O_2max_ during repeated human testing with their setup. After accounting for technological error, they concluded that over 90% of this variability is due to biological factors, with less than 10% coming from technological sources, a finding similar to Armstrong and Costill [46]. In their study, Katch and colleagues [45] determined technological error by calculating the standard deviation of V̇O_2_ from 20 samples fed through a gas analyzer at a standard flow rate. However, it is unclear whether all samples were from a single test (thus having similar gas composition), or if this process was repeated for different tests. The use of a single gas composition would likely decrease variability compared to repeated analyzing of different gas compositions. Additionally, the study was limited to one flow rate and was conducted in the early 1980s using gas analyzers and flow meters that may not be comparable to modern sensors. Furthermore, independent analysis of gas concentration and flow underestimates technological errors compared to their combined assessment. Similar limitations affect the generalizability of other studies attempting to differentiate between biological and technological variability [46, 47]. Understanding the relative contribution of technological and biological variability to repeated human testing provides important insights into how more reliable equipment can reduce measurement variability. The repeated simulation and human exercise testing in this study allowed us to distinguish between technological and biological variability. Therefore, the present study also aims to determine the biological and technological variability of contemporary CPET systems.

## 2 Methods

### 2.1 General Study Design

This study consisted of two parts: (1) validation of metabolic analyzers during simulated exercise testing and (2) verification and comparison during steady‐state cycling in well‐trained human participants. All measurements were performed on the same day and repeated the next day to assess between‐day reliability.

### 2.2 Equipment

CPET data were collected using five popular CPET systems (Table 1). Four of these systems have been examined in a previous study [18], but one system (KORR Medical Technologies, Salt Lake City, UT) was not included earlier due to cost and time constraints of the manufacturer. In this study, this system was available, with the manufacturer present to ensure proper device application and handling. The manufacturers of the CPET systems or the metabolic simulator had no role in the study design, data analysis, interpretation, or the decision to submit the paper for publication.

**TABLE 1:** Software and hardware specifications for CPET system.

	Vyntus CPX	Oxycon Pro	VO2 Master Pro	Calibre	KORR
Manufacturer	Jaeger Medical, Chicago, USA	Jaeger medical, Chicago, USA	VO2 Master Health Sensors Inc., Vernon, BC, Canada	Calibre Biometrics, Wellesley, MA, USA	Korr Medical Technologies, Salt Lake City, Utah
Type	Mixing‐chamber (not tested) and breath‐by‐breath	Mixing‐chamber and breath‐by‐breath	Breath‐by‐breath	Breath‐by‐breath	Mixing‐chamber
Volume measurement	Digital volume transducer (DVT) (Jaeger, Chicago, USA)	DVT (Jaeger, Chicago, USA)	Differential pressure (VO2 Master Pro)	Differential pressure (Sensirion, Stäfa, Switzerland)	Differential Pressure (Amphenol Allsensors, Morgan Hill, CA USA)
O_2_ measurement	Chemical fuel cell (Teledyne, CA, USA)	Chemical fuel cell (Teledyne, CA, USA)	Chemical fuel cell (Envitec NJ, USA)	Electro chemical (Angst Pfister, Switzerland)	Chemical fuel cell (Teledyne, CA, USA)
CO_2_ measurement	Non‐Dispersive Infrared (Jaeger, Chicago, USA)	Non‐Dispersive Infrared (Jaeger, Chicago, USA)	N/A	Thermal conductivity (Sensirion, Switzerland)	Non‐Dispersive Infrared (Phillips, Wallingford CT, USA)
Accuracy for volume, V̇O_2_, V̇CO_2_	±3% (50 mL) for all outcomes	±3% (50 mL) for all outcomes	Not stated	Not stated	±3% (50 mL) for all outcomes
Approximate system cost^a^	€32 000^b^	N/A	€6900	€465	€12 967^b^

Abbreviations: CO_2_ = carbon dioxide; O_2_ = oxygen; V̇CO_2_ = carbon dioxide production; V̇E = minute ventilation; V̇O_2_ = oxygen uptake.

^a^ Cost for a system in The Netherlands in 2025, exclusive of shipping costs. Note that the cost for most systems is dependent on the configurations (e.g., with or without ECG add‐on).

^b^ Exclusive of local taxes.

### 2.3 Metabolic Simulator

The human gas exchange response during exercise was mimicked using a state‐of‐the‐art metabolic simulator consisting of a piston pump breathing simulator combined with a gas‐infusion system (Relitech Systems BV, Nijkerk, The Netherlands). This system is reliable and produces highly accurate breath‐by‐breath variables [18, 28]. A detailed description of the simulator can be found elsewhere [18, 28], and a concise description will thus be provided here. Briefly, the breathing simulator uses a motorized syringe (piston) to simulate breathing variables (tidal volume, breathing frequency) and can also simulate different breath gas concentrations by pumping room air back and forth and injecting amounts of pure CO_2_ and N_2_ (purity ≥ 99.99%; Linde Gas, Netherlands). The injection of 100% CO_2_ creates a gas that simulates a precise amount of V̇CO_2_ at different breathing frequencies, while 100% N_2_ dilutes the ambient air O_2_ to a specific O_2_ concentration to simulate V̇O_2_ rates. The simulated V̇O_2_ and V̇CO_2_ are automatically calculated as detailed previously [18].

The ratio of V̇CO_2_ to V̇O_2_ (i.e., RER) can also be adjusted to range between 0.75 and 1.05. The amount of CO_2_ and N_2_ injected during each exhaled breath by the metabolic simulator is controlled by high‐precision mass flow controllers (Bronkhorst High‐Tech B.V., Ruurlo, The Netherlands), achieving a precision of less than 0.2% for the simulated O_2_ and CO_2_. Coupled with the simulator's volume stroke accuracy, this setup produces V̇O_2_ and V̇CO_2_ with an accuracy of < 0.5%, even at high V̇E. The simulator was certified 6 months before the first test day and again 4 months after the last simulation test day.

### 2.4 Simulation Protocol

The CPET systems were connected directly to the outlet of the metabolic stimulator, using custom‐made adaptors when needed. We aimed to use the same dead space for all systems and to reduce turbulence caused by the custom adaptors. Each CPET system followed a standardized procedure to evaluate V̇E, BF, V̇O_2_, V̇CO_2_, and RER as primary outcomes. Additional data included FiO_2_, FiCO_2_ (the percentage of oxygen and carbon dioxide in inspired air, respectively), and FeO_2_, FeCO_2_ (the percentage of oxygen and carbon dioxide in expired air, respectively). Note that not all systems measured or provided this data. The mixing chamber method in Oxycon Pro and KORR does not measure FiO_2_ and FiCO_2_ continuously (but only at the start of a measurement), Calibre does not include these variables in its time and breath table data output, and VO2 Master Pro only provides mixed FeO_2_ values.

The “Std” mode procedure on the simulator was used first, with the tidal volume set at 2 L and RER at 1.00 (V̇O_2_, V̇CO_2_ equal). During this mode, BF changed from 20 to 40, 60, and 80 min^−1^. V̇O_2_ and V̇CO_2_ at each BF were 1, 2, 3, and 4 L min^−1^. The BF's and tidal volume used mimic physiological values reported during human physical activity and exercise testing [40, 48, 49, 50, 51]. For instance, while the maximum tidal volume is slightly lower than the maximum tidal volume reported in the literature for well‐trained athletes (3 vs. ~3.8 L min^−1^), the BF is higher (80 vs. ~65 breaths min^−1^), and the resulting V̇E is only slightly lower (240 vs. ~250 L min^−1^) as reported in literature [48, 49, 50, 51].

A second protocol was performed in “CPX” mode procedure to simulate different combinations of RERs with increasing BFs and V̇E. The RER variations were used to mimic increased carbohydrate oxidation with higher exercise intensities and to simulate buffering of ion concentrations [H^+^] by bicarbonate [HCO_3_^−^] at very high exercise intensities [52]. The simulated RER values were 0.75, 0.85, 0.95, and 1.05, with V̇O_2_ at 1, 2, 3, and 4 L min^−1^ at each RER, corresponding to V̇CO_2_ of 0.75, 1.7, 2.85, and 4.2 L min^−1^. Note that the lowest step of the CPX procedure (i.e., with BF of 10 min^−1^, RER 0.75, and V̇O_2_ of 1 L min^−1^) required a separate mask connector (type R) with volume calibration procedure as recommended for VO2 Master Pro. This stage was simulated separately while using the R‐mask adapter. Further, the maximum breathing frequency for KORR is 65 breaths min^−1^ and the last stage of the Std protocol was therefore not used in the analysis for this system.

For breath‐by‐breath systems, each stage lasted at least 2 min to ensure sufficient time for breath collection stabilization, and the graphical user interface for each system was checked to confirm a steady state. For mixing chamber systems, each step was simulated for approximately 5 min to allow sufficient time for mixing chamber systems to flush the mixing chamber, as verified by visual inspection of the graphical user interface.

Between‐day reliability for all systems was measured by repeating the same simulation experiments on the following day at roughly the same time, always before the human experiments to minimize any potential impact of residual humidity from the human testing on the results [53].

### 2.5 Human Validation Protocol

Human exercise was used to verify the results obtained during the simulation tests, as described previously [18]. Briefly, three to five days before the experiments, each participant performed a graded maximal exercise test to determine their gas exchange/first ventilatory threshold. During the experiments, a total of six well‐trained healthy males (mean ± SD age 28.0 ± 5.8 years, body mass 73.3 ± 6.6 kg, V̇O_2peak_ 63.8 ± 8.0 mL kg^−1^ min^−1^), then cycled at the highest intensity at which physiological variables remained stable (i.e., 5% below their gas exchange/first ventilatory threshold; 163 ± 32 W; 2.3 ± 0.4 W kg^−1^) while gas exchange data were collected two times per system for three (breath‐by‐breath) or five (mixing chamber) minutes in a randomized and counterbalanced order. The same experiment was repeated 1 day later to assess the between‐day reliability.

After analyzing the results of the first human experiments, it was observed that Calibre had relatively large errors relative to the reference range. Therefore, after contact with the manufacturer to discuss possible causes, a series of tests was performed to find possible causes of this observation. To this purpose, two additional rounds of tests were performed among three and four individuals (all the same individuals as the first test), respectively, using a similar methodology to the first test.

### 2.6 Data Collection Settings for Each CPET System

The metabolic simulator mimics human breathing and creates artificial, highly accurate, known breaths. The mass‐flow controllers used in the metabolic simulator for CO_2_ and N_2_ have a temperature‐controlled output normalized to absolute volume output in standard temperature and pressure dry (STPD) (SLN, normalized standard liters), as detailed previously [18]. V̇E, the volume strokes from the piston pump of the metabolic simulator, uses room air, and is thus at ambient conditions (ambient temperature and pressure; ATP).

CPET systems are typically used for human testing. Human expired volumes have a higher temperature and humidity than ambient air; expired volumes are thus always expressed in saturated body temperature and pressure conditions (BTPS). By measuring or assuming a specific humidity, temperature, and pressure of the expired air, the CPET systems convert the values measured in BTPS to STPD for V̇O_2_ and V̇CO_2_ to allow comparison between different measurement conditions. For example, CPET breath‐by‐breath systems typically assume the expired gas is 100% humid and has a temperature of 31.5°C. Since this assumption is incorrect during the metabolic simulation experiments, the gas volumes in STPD require correction to allow comparison with the simulated values. We therefore turned off the BTPS correction within the software application when possible. The firmware updates made on the Calibre device since our previous publication did not allow assessment of the Calibre against a metabolic simulator. Therefore, the simulation was not performed on this device.

Room temperature and relative humidity (Inkbird, PTH‐9CW) ranged between 19°C–21°C and 45%–67%, respectively, during all simulation and cycling measurements. During all experiments, the lab was well ventilated by opening windows and doors.

### 2.7 CPET Calibration

Each CPET system was calibrated according to the manufacturer's guidelines before the simulations, and again before the human experiments. All manufacturers used their own gas for calibration to best reflect typical system calibration. The volume for all systems was calibrated manually using a certified 3 L syringe from each respective manufacturer, or using the automated method within the system (Vyntus CPX and Oxycon Pro).

### 2.8 Data Processing

During the simulation tests, the mean value of the last minute of each stage was used for analysis to ensure adequate flushing of the gas‐filled dead space of the simulator. The period selected for analysis was also confirmed by visual inspection of a steady state.

Data processing for the human cycling experiments was done as detailed previously [18]. Briefly, data were analyzed over the final minute of each period and subsequently averaged over the two counterbalanced 1‐min periods to make comparisons between systems. Because a true reference is not available in human testing (because the actual gas exchange values by each participant are unknown), we computed reference values based on the average V̇O_2_ and V̇CO_2_ values recorded by Vyntus CPX and Oxycon Pro while correcting their measured values for the respective errors in V̇O_2_ and V̇CO_2_ determined in the simulation experiments. Vyntus CPX and Oxycon Pro were used to calculate the reference value because these systems have previously demonstrated relatively high accuracy and reliability in both simulation and human experiments [18, 28, 33]. The computation of a reference value based on these systems therefore provides the most accurate indication of the expected true value. During the additional tests with Calibre, only the Vyntus CPX device was used as a reference.

### 2.9 Statistical Analysis

The accuracy of the CPET systems was assessed for the main ventilatory and gas exchange variables: V̇E (L min^−1^), BF (breaths min^−1^), V̇O_2_ (mL min^−1^), V̇CO_2_ (mL min^−1^), and RER. For the trials with RER < 1.00 (metabolic simulator in “CPX” mode), we also computed and compared the energy expenditure derived from fats and carbohydrates and total energy expenditure from the simulated and measured V̇O_2_ and V̇CO_2_ using Jeukendrup's equation for moderate‐ to high‐intensity exercise [52]. This was done to determine the impact of errors in the measured V̇O_2_ and V̇CO_2_ values on the estimation of substrate and energy expenditure.

Agreement between the CPET systems and the metabolic simulator was assessed in several ways. First, the measurement error was calculated for the simulation test by subtracting the expected value (i.e., simulated) from the measured value (i.e., converted CPET readouts). We expressed this error as a percentage of the expected value (i.e., [(measured—expected)/expected] × 100) and computed the average relative percentage error and average absolute percentage error (AAPE; where all values are positive) for all simulation steps for each system to indicate the overall measurement error. To assess if the relative (i.e., non‐absolute) error changed with higher simulated values, we assessed if the slope of the regression line fitted on the error differed significantly from zero.

To objectively assess the agreement between the simulator and CPET systems, we used a statistical approach proposed by Shieh [54] with the percentage difference as the unit for comparison as detailed previously [18]. In this method the mean difference and variability of the difference between the simulator and CPET system is assessed in relation to an a priori determined threshold. Error thresholds for the main ventilatory and gas exchange variables, as well as energy expenditure, were considered: good, < 3%, acceptable, < 5%, and poor ≥ 5%. For substrate use errors were rated < 5% as good, < 10% as acceptable, and ≥ 10% as poor.

Between‐day (absolute) technological variability was quantified by calculating the standard deviation of the difference between the measured values per day for each step of the simulation. This standard deviation was subsequently divided by the mean of the measurements, multiplied by 100, to express it as a percentage (i.e., coefficient of variation). Similarly, biological variability was quantified as the standard deviation of the difference between the measured values for each day of the human experiments and subsequently expressed as a percentage. The coefficient of variation computed over the repeated simulation testing was subsequently expressed as a percentage of the coefficient of variation computed over the repeated human testing to determine the contribution of technological variability and biological variability to repeated human exercise testing.

## 3 Results

### 3.1 Metabolic Simulation

All data, including errors in original units, as well as both relative and absolute percentage errors for all individual simulation steps, are available in the Appendix S1.

**FIGURE 1:**
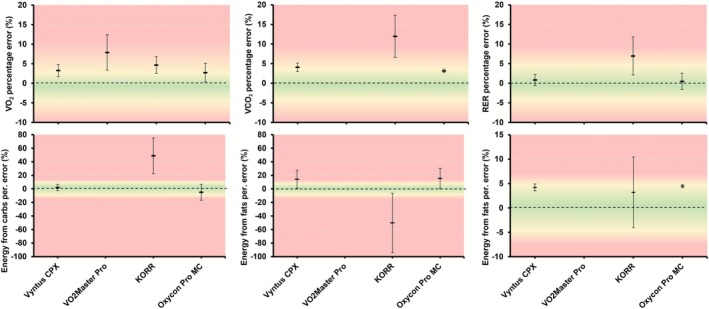
Mean ± standard deviation relative percentage errors for each device for V̇O_2_, V̇CO_2_, RER, energy derived from carbohydrates, energy derived from fats, and total energy expenditure. Horizontal lines represent the average error over all simulated steps over both days, while error bars represent the standard deviation of the error over all simulated steps of both days. Wider error bars indicate a lower precision of the measured variable. No error for substrate usage or total energy expenditure is available for VO2 Master Pro as this device measures only V̇O_2_. RER, respiratory exchange ratio; V̇CO_2_, carbon dioxide production; V̇O_2_, oxygen uptake.

**FIGURE 2:**
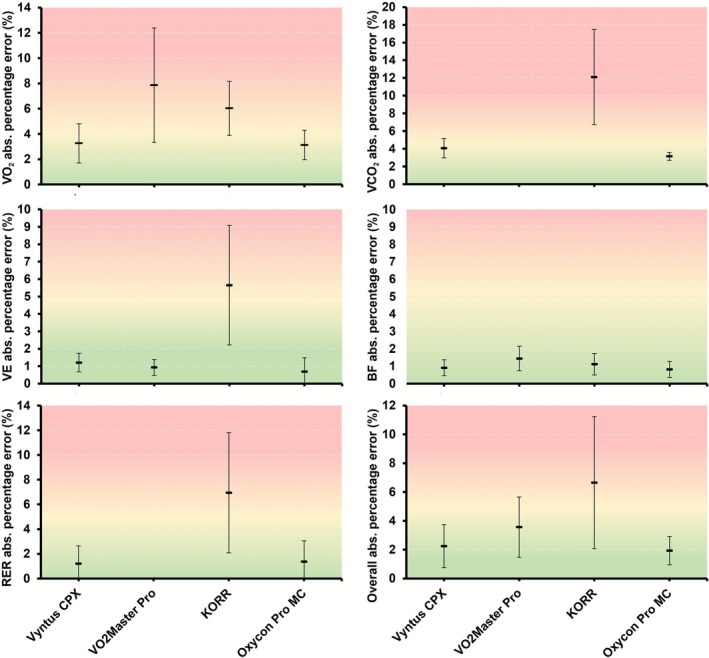
Mean ± standard deviation of absolute percentage errors for gas exchange variables per device. Horizontal lines depict the mean error over all simulated steps of both days, while error bars represent the standard deviation of the error. No error for V̇CO_2_ or RER is available for VO2 Master Pro as this device measures only V̇O_2_. The overall percentage error is computed over all gas exchange variables in the figure. BF, breathing frequency; RER, respiratory exchange ratio; V̇CO_2_, carbon dioxide production; V̇E, minute ventilation; V̇O_2_, oxygen uptake.

Average relative percentage errors across all simulated volumes are shown in Table 2 (respiratory variables) and Table 3 (energy expenditure variables), and are also illustrated in Figure 1. The absolute percentage error for V̇E, BF, V̇O_2_, V̇CO_2_, RER, and the overall error for each device, averaged over all simulated steps on both days, are reported in Table S1 and illustrated in Figure 2. Table S2 presents the absolute percentage errors for energy from fats, carbohydrates, and total energy expenditure, averaged across all simulated steps, while Figure S1 visualizes these errors.

**TABLE 2:** Mean ± SD relative percentage errors (%e) for respiratory variables, averaged over all simulated steps of both days.

System	%e V̇E	%e BF	%e V̇O_2_	%e V̇CO_2_	%e RER
Vyntus CPX	1.19 ± 0.55**	−0.63 ± 0.77**	3.26 ± 1.55	4.07 ± 1.08	0.82 ± 1.46
Oxycon Pro MC	−0.08 ± 1.33*	−0.82 ± 0.46**	2.72 ± 2.38	3.15 ± 0.44	0.46 ± 2.07
KORR	2.88 ± 3.43	−0.96 ± 0.70	4.66 ± 2.14	11.9 ± 5.37	6.94 ± 4.87
VO2 Master Pro	0.61 ± 0.74**	−1.44 ± 0.70*	7.86 ± 4.53	—	—

*Note:* **Good agreement (< 3% error); *acceptable agreement (< 5% error); No star indicates that we were unable to establish good or acceptable agreement.

Abbreviations: BF, breathing frequency; RER, respiratory exchange ratio; V̇CO_2_, carbon dioxide production; V̇E, minute ventilation; V̇O_2_, oxygen uptake.

**TABLE 3:** Mean ± standard deviation relative percentage errors (%e) for substrate use and total energy expenditure, averaged over all simulated steps of both days.

System	%e Energy from carbs	%e Energy from fats	%e Total energy expenditure
Vyntus CPX	1.99 ± 4.75	14.1 ± 13.4	4.21 ± 0.71*
Oxycon Pro MC	−4.91 ± 11.8	15.3 ± 15.2	4.46 ± 0.32*
KORR	48.8 ± 26.4	−50.3 ± 43.8	3.19 ± 7.23
VO2 Master Pro	n.a.	n.a.	n.a.

*Note:* **Good agreement (< 5% error); *acceptable agreement (< 10% error); No star indicates that we were unable to establish good or acceptable agreement.

**FIGURE 3:**
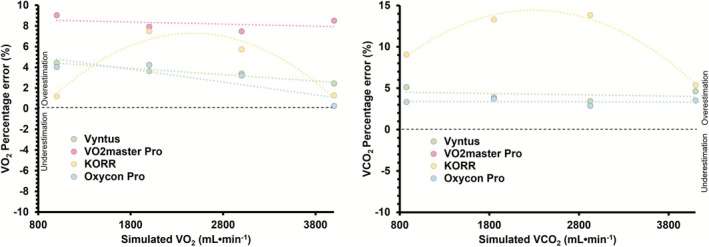
Relative percentage error for V̇O_2_ (left) and V̇CO_2_ (right), as a function of the simulated V̇O_2_ and V̇CO_2_ for each device. Errors are averaged over each step of the “Std” (i.e., RER = 1.00) and “CPX” (i.e., RER increases with increased V̇O_2_) protocols for both days. V̇CO_2_, carbon dioxide production; V̇O_2_, oxygen uptake.

The relative percentage error varied significantly with higher simulated volumes for some devices, while remaining constant for others (Figure 3 and Table S3). Between‐day reliability is reported in Table 4 in both original and percentage units (coefficient of variation).

**TABLE 4:** Between‐day technological variability as determined during simulation testing in original and percentage units.

System	V̇E (L min^−1^)	BF (breaths min^−1^)	V̇O_2_ (mL min^−1^)	V̇CO_2_ (mL min^−1^)	RER	Energy from carbs (Kcal min^−1^)	Energy from fats (Kcal min^−1^)	Total energy expenditure (Kcal min^−1^)
Original units								
Vyntus CPX	±0.60	±0.02	±30.7	±35.6	±0.01	±0.14	±0.09	±0.07
Oxycon Pro MC	±0.25	±0.08	±11.6	±17.9	±0.01	±0.19	±0.19	±0.02
KORR	±3.16	±0.17	±81.7	±136	±0.04	±0.26	±0.29	±0.54
VO2 Master	±0.39	±0.06	±81.5	—	—	—	—	—

	V̇E (%)	BF (%)	V̇O_2_ (%)	V̇CO_2_ (%)	RER	Energy from carbs (%)	Energy from fats (%)	Total energy expenditure (%)
Percentage units (CV)								
Vyntus CPX	±0.72	±0.06	±1.19	±1.40	±1.42	±14.1	±7.64	±0.57
Oxycon Pro MC	±0.30	±0.20	±0.46	±0.71	±0.56	±20.1	±14.0	±0.14
KORR	±3.71	±0.45	±3.15	±4.99	±4.28	±21.1	±18.8	±4.54
VO2 Master	±0.48	±0.14	±3.01	—	—	—	—	—

Abbreviations: BF, breathing frequency; MC, mixing chamber; RER, respiratory exchange ratio; V̇CO_2_, carbon dioxide production; V̇E, minute ventilation; V̇O_2_, oxygen uptake.

### 3.2 Human Validation

Six well‐trained male participants volunteered to participate, but only four participants completed all three test sessions (V̇O_2peak_ assessment and two test–retest days) and were therefore included in the analysis. Mean ± SD characteristics were an age of 29.3 ± 7.09 years, a body mass of 73.6 ± 7.65 kg, a V̇O_2peak_ of 62.0 ± 7.26 mL kg min^−1^, and a work rate at first ventilatory threshold of 169 ± 42.5 W.

**FIGURE 4:**
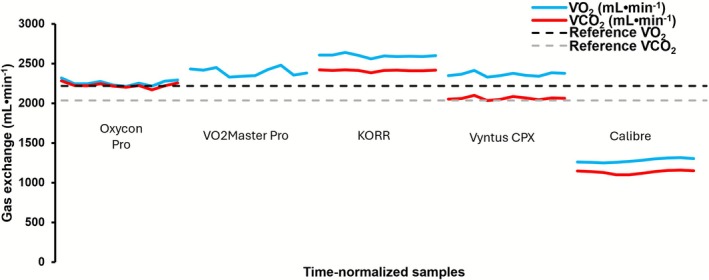
Mean ± SD measured V̇O_2_ and V̇CO_2_ during the cycling experiments of both days for each system. All V̇O_2_ and V̇CO_2_ values were first averaged over the two counterbalanced trials within each subject per day, then averaged over both days, and then averaged over all participants. For all tests, reference values were calculated as specified in section 2.8. V̇CO_2_, carbon dioxide production; V̇O_2_, oxygen uptake.

The mean heart rate was very similar between all measured devices (≤ 2 beats min^−1^ difference) and did not differ significantly (Table S4). The measured gas exchange variables, substrate use, and energy expenditure measured during the cycling experiments is reported in Table S5. Figure 4 also shows the V̇O_2_ and V̇CO_2_ measured by each system during the cycling experiments. Finally, Table S6 reports the between‐day reliability in original and percentage units from the human experiments.

## 4 Discussion

The primary aims of this study were to investigate: (a) the accuracy of five different CPET devices under metabolic simulation and human cycling conditions, and (b) the between‐day reliability of these systems during both simulation and human exercise conditions. The most important findings of this study are that accuracy during metabolic simulation varied substantially between the different systems, with overall relative percentage errors for V̇O_2_ ranging from 2.7% to 7.9%. During human testing, the differences in the measured V̇O_2_ relative to the reference (expected) values were even larger, with a range of −40% to 17%. Comparable ranges were found for V̇CO_2_ relative percentage errors.

Between‐day technological variability for V̇O_2_ as determined during repeated metabolic simulation experiments ranged from 0.5% to 3.2%, with comparable values (0.7% to 5.0%) reported for V̇CO_2_. During human testing, the between‐day variability (technological plus biological) for V̇O_2_ and V̇CO_2_ ranged from 3% to 12% and 3% to 6%, respectively, when excluding one highly variable system (Calibre, with CV of ~79%). During repeated human exercise testing, biological variability accounted for approximately 60% to 70% of the overall test–retest variability in V̇O_2_ and V̇CO_2_, respectively, while technological variability accounted for approximately 40% to 30% of the between‐day variability in V̇O_2_ and V̇CO_2_. The following sections discuss each of these findings in more detail.

### 4.1 Validity During Metabolic Simulation

The metabolic simulator allowed us to compare the simulated gas exchange values to those measured by each system. Using this approach, we found a relatively large range in the accuracy of respiratory gas exchange values (Table 2 and Table S1, Figures 1 and 2). First, the generally relatively high accuracy for Vyntus CPX and the Oxycon Pro confirms previous research showing generally high validity during (simulated) exercise [18, 28]. Notably, the absolute percentage errors in V̇O_2_ and V̇CO_2_ for both devices were approximately 0.2%–1.0% larger in the present study compared to the same device assessed in our previous study against the same simulator [18]. This small increase in error may simply reflect variability associated with repeated testing, but it might also indicate degradation of some sensors over time (~1.5 years between the current study and the previous). In partial support of the latter interpretation, the O_2_ sensor for Vyntus CPX was replaced just several weeks after the experiments. When this slight increase in error was combined with the variability in error during each simulated step, it resulted in an absence of statistically significant agreement at the 3% or 5% thresholds for V̇O_2_ and V̇CO_2_ (Table 2 and Table S1). In other words, while on average the errors were below these thresholds, the variability in error was too high to conclude that this error would generally be within this threshold. However, statistically significant agreement was achieved for other outcomes such as V̇E, breathing frequency, and RER.

KORR has not previously been assessed against a metabolic simulator. With absolute percentage errors of 6% and 12% for V̇O_2_ and V̇CO_2_, respectively (Table S1), this device performed relatively modestly and poorly compared to other devices in the present study (Table S1) and our previous study using the same methodology [18]. This was largely due to a relatively high error in the measured V̇E (5.7%; Table S1), which in turn resulted in errors in V̇O_2_ and V̇CO_2_. This is further supported by the relatively similar values for fractions of expired oxygen and carbon dioxide between KORR and the other devices that provided these values (Appendix S1). The differential pressure sensor used for volume measurement in KORR is not inherently prone to error, as other devices in the present study and our previous study have demonstrated high volume accuracy using a similar sensor type. Instead, the error may stem from how this sensor data is filtered and used in subsequent computations, or from errors in the sensor's calibration. Additionally, while the V̇O_2_ and V̇CO_2_ achieved steady‐state values on the graphical user interface during testing, the output received contained only a few data points per simulation step, which may nevertheless have introduced inaccuracies.

VO2 Master Pro showed a higher accuracy in the present metabolic simulator experiments as compared to our previous study [18]. Specifically, the overall absolute percentage error for V̇O_2_ was approximately half the error observed in our previous study (7.9% vs. 13.1%, respectively), with the direction now also being positive (overestimation by ~7.8%) whereas it was negative (underestimation by 11.7%) in the previous study. Because the simulator and hardware of the CPET system were similar in the present and our prior study [18], the differences most likely reflect changes in the algorithms for measuring gas exchange variables, or algorithms for compatibility with the metabolic simulator. Specifically, the new software exhibited a “metabolic simulator mode”, thus enabling direct comparison with the simulator output. Although we previously corrected for differences between the metabolic simulated output and CPET systems measured outputs, the direct comparison may nevertheless have improved accuracy and therefore likely better reflects the actual performance of the device. The findings of the present study are in line with those of another recent study that showed relatively high accuracy of VO2 Master when assessed against a metabolic simulator (absolute percentage error of ~2.5% for V̇O_2_) [44]. Although Thiessen and colleagues [44] used a different brand metabolic simulator (Vacumed), it has previously been shown that both the Vacumed and Relitech simulator (the latter used in the present study) provide nearly identical simulated values when assessed by the same CPET system [28]. The higher absolute percentage error for V̇O_2_ in the present study as compared to Thiessen and colleagues [44] may therefore primarily reflect different approaches to how the device was used during simulation measurements. Specifically, Thiessen and colleagues [44] observed that the relative humidity, temperature, and barometric pressure registered by the metabolic simulator were not identical to those of VO2 Master and therefore used the values measured by the simulator to derive correction factors. Such an approach will reduce differences between the simulator and CPET system assessed. However, we believe that the differences potentially caused by variations in humidity, temperature, and pressure measurement are integral to the CPET system and should therefore not be imputed with data from the metabolic simulator. Indeed, during the normal application of human testing, the humidity, temperature, and pressure measured by the device are used to derive correction factors to subsequently convert the measured V̇O_2_ and V̇CO_2_ from ATP to STPD (to allow comparison between different conditions). Errors in the measured humidity, temperature, and pressure will therefore also affect the resulting V̇O_2_ and V̇CO_2_ during human testing and should thus be considered an integral component of the CPET system, similar to the volume or O_2_ measurement. In further support of this, the approach we used (i.e., utilizing the CPET's humidity, temperature, and pressure values) yielded a relative percentage error from simulation testing that closely matched the error observed relative to the reference value during human testing (7.86% vs. 8.37%, respectively). If we had instead used the humidity, temperature, and pressure values from the simulator, we could have artificially increased the accuracy during metabolic simulation, which would not have matched the accuracy during human testing. Nevertheless, the resulting absolute percentage error of 7.9% for V̇O_2_ in the present study is in line with several of the devices investigated in our previous study [18], and suggests potential for V̇O_2_ assessment with this device.

As detailed in the methods, the firmware updates made on the Calibre device since our previous publication did not allow assessment of the Calibre against a metabolic simulator. Therefore the simulation was not performed on this device.

#### 4.1.1 Error for Substrate Use and Total Energy Expenditure

Errors for substrate use were substantially larger than the errors for gas exchange outcomes (Table 3, Figure 2). This observation is consistent with previous research [18] and implies that small errors in gas exchange can result in significant differences in the computed substrate use. The errors were particularly large for KORR, although they were also still substantial (~15%) for Vyntus CPX and Oxycon Pro. Carbohydrate utilization accuracy is particularly sensitive to accurate determination of V̇CO_2_ because the carbohydrate oxidation equation used (4.210 × V̇CO_2_ −2.962 × V̇O_2_) places greater weight on V̇CO_2_ than V̇O_2_. Care should therefore be taken when using substrate outcomes from CPET systems for nutritional or training advice. In contrast, total energy expenditure was measured more accurately, with all systems showing relative errors < 5% on average. The smaller error in total energy expenditure as compared to substrate use results from an underestimation in energy derived from one substrate being associated with an increase in the energy derived from the other substrate, thereby leading to a smaller net error in the estimated total energy expenditure.

#### 4.1.2 Change in Error With Changes in Flow During Metabolic Simulation

Measurement error may increase with higher flow rate due to limitations in sensor response or linearisation [18, 22, 27, 55]. We therefore explored if the error in V̇O_2_ and V̇CO_2_ changed with higher simulated V̇O_2_ and V̇CO_2_ (Figure 3, Table S3). This analysis showed three out of four systems to exhibit no significant change in either V̇O_2_ or V̇CO_2_ relative error with higher V̇O_2_ and V̇CO_2_, which gives some confidence to using these systems, for example, elite athletes that may achieve values beyond those assessed in the present study. While not significant when assessed using a linear relationship, KORR, however, visually appeared to exhibit a parabolic change in error, with lower error at both low and high simulated V̇O_2_ and V̇CO_2_ values, and higher errors at intermediate values (Figure 3).

### 4.2 Validity During Human Exercise Testing

Like the metabolic simulation testing, human testing also showed a wide range of errors in the measured outcomes compared to the reference (expected/true) values (Table S5, Figure 4). While the errors during human testing approximately reflected the magnitude and direction observed in the simulator testing, the differences did not match exactly. This was expected because human testing is influenced not only by technological measurement error but also by human biological variability, which makes it harder to determine the true error of each system [26, 44, 45]. Nonetheless, the general similarity confirms that the simulation test findings can be applied to real human exercise. For example, during human testing KORR also showed relatively larger deviations, similar to the simulation assessments.

**FIGURE 5:**
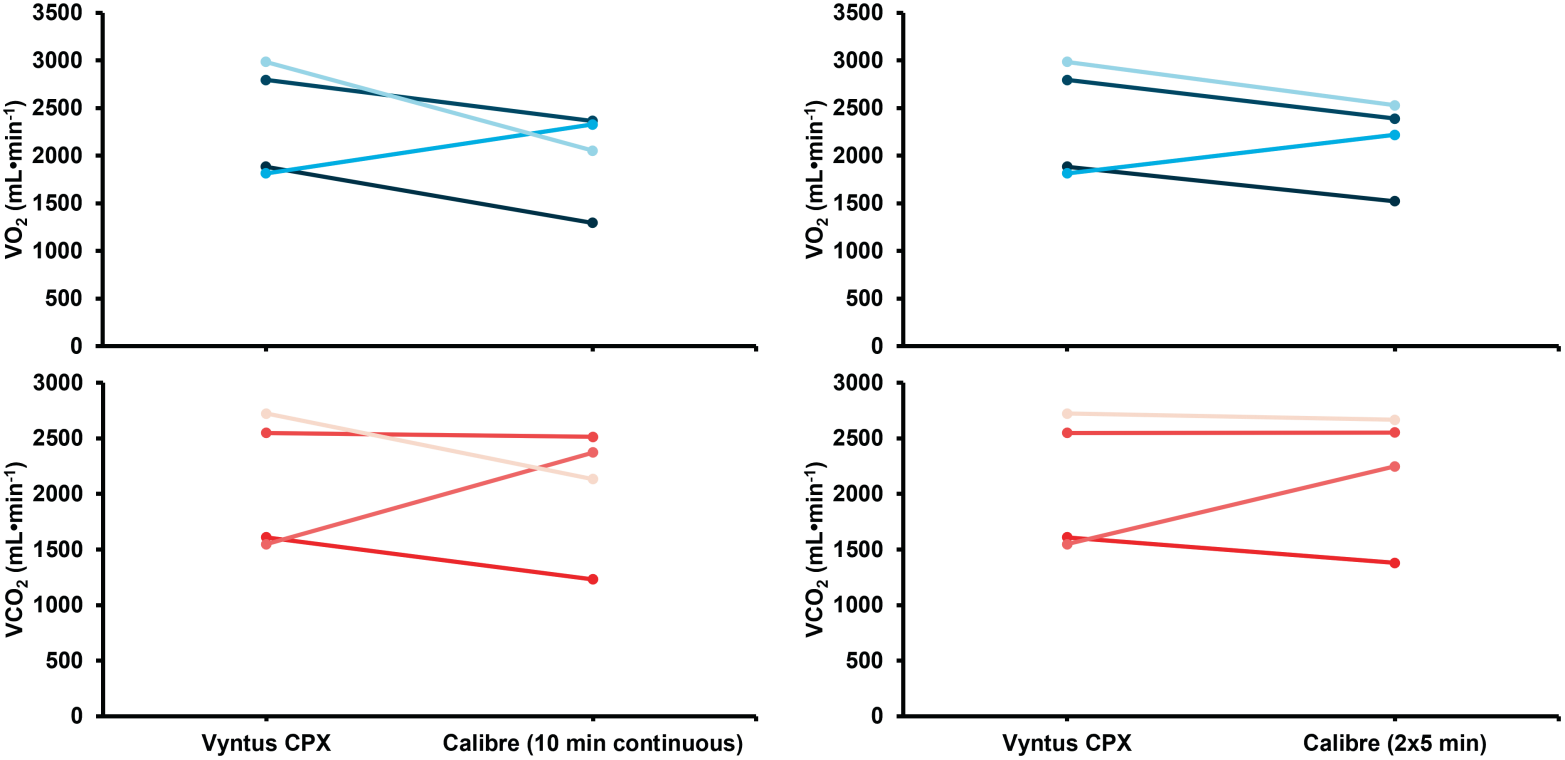
Gas exchange data from the second Calibre retest. Each line depicts the average V̇O_2_ (blue) or V̇CO_2_ (red) for one participant across two days as measured by Vyntus CPX or Calibre, with the left panels depicting the results obtained with a 10‐min continuous Calibre recording, and the right panels depicting the results obtained with a 2 × 5 min discontinuous recording.

Interestingly, Calibre showed a very large deviation in both V̇O_2_ and V̇CO_2_ during human exercise relative to the reference values (Figure 4), despite strictly adhering to the manufacturer's guidelines for using the device. This finding contrasts previous findings where high agreement was observed between Calibre and other CPET systems during human exercise using a similar methodological set‐up [18]. After contact with the manufacturer to discuss possible causes, an additional round of testing was performed among three individuals (all the same individuals as the first test‐round) with a newly provided device. This second round aimed to assess if the accuracy improved when device calibration was performed immediately prior to the measurement as opposed to 2–3 min beforehand. Despite this modification and new device, performance remained highly variable (Figure S2; Table S7), with reasonable accuracy during the first day of testing, but very poor performance during the repeated tests on the second day –despite using identical procedures and similar participants–. Because V̇E was similar (Table S7), these errors likely originated from errors in the measured gas concentrations due to incorrect baseline correction of the sensors. The manufacturer suggested that the time interval between the first two participants (~50 min) may have contributed to vapour accumulation within the device, potentially leading to permanent sensor damage that in turn explained the poor performance during Day 2. To test this possibility, we conducted an additional round of testing with four participants, incorporating (a) longer rest intervals between measurements (up to 3 h), (b) calibration immediately prior to each measurement, and (c) both a continuous 10‐min measurement and a counterbalanced 2 × 5 min measurement (with the continuous and counterbalanced measurements performed using a separate device) to evaluate whether the counterbalancing procedure contributed to the poorer performance. Moreover, a representative from Calibre was present to observe all tests performed on both days and ensure proper operational handling of the device. Although the performance improved relative to the prior tests, the findings from these experiments still showed relatively large differences compared with the Vyntus CPX (average absolute percentage errors > 20% for both V̇O_2_ and V̇CO_2_ across all participants and both protocols/devices; Figure 5; Table S7). Moreover, the V̇O_2_ and V̇CO_2_ measured by Calibre were, respectively, 6‐ and 17‐fold more variable between testing days than the V̇O_2_ and V̇CO_2_ measured by Vyntus CPX, indicating substantial inconsistency. The observed differences and inconsistent results are likely due to the Calibre architecture's sensitivity to firmware, particularly in how it converts sensor readings into actual breath measurements. Specifically, firmware changes intended to improve accuracy in one measurement domain (e.g., V̇O_2 Max_) may have inadvertently increased error in another domain (exercise < VT1). In addition, sensor drift or offset, changes in environmental conditions, and imperfect mask fit (the latter evidenced by V̇E differences > 13%) may further explain these observations. Therefore, these findings indicate that caution is warranted when using this device for the assessment of gas exchange during exercise.

### 4.3 Between‐Day Variability During Repeated Metabolic Simulation (Technological Reliability)

In some situations, lower validity of CPET outcomes may be acceptable if the outcomes can be measured reliably (i.e., with low variability in repeated measures of the same simulated value). The typical variation (CV) during repeated simulation testing also ranged substantially between systems, and between outcomes, from as low as 0.06% for breathing frequency in VO2 Master up to 21% for energy derived from carbohydrates for KORR (Table 4). Even for generally highly accurate systems (Vyntus CPX and Oxycon Pro), the typical variability in energy derived from substrates ranged between ~8% and 20%, suggesting that substantial changes in substrate use are required in repeated testing before these exceed the technological measurement variability. The between‐session variability for V̇O_2_ and V̇CO_2_ ranged from 0.5% to 3.12% and from 0.7% to 5.0%, respectively (Table 4). These findings have important implications for studies that aim to assess, for example, small differences in V̇O_2_ or energy expenditure between conditions or individuals. For example, differences in running economy between different shoes are typically small (< 4% [6, 56, 57]). While repeated measures have been advocated to reduce measurement error [58], our findings indicate that the use of reliable CPET systems also has the potential to contribute substantially to increasing the ability to detect these small effects.

### 4.4 Between‐Day Variability During Repeated Human Exercise Testing (Biological and Technological Reliability)

The between‐day variability during human exercise testing was 3–4 times higher (i.e., median CV of 7.5% and 6.4% for V̇O_2_ and V̇CO_2_, respectively; Table S6) than the variability during simulated exercise (CV of ~1.5%–2% for both V̇O_2_ and V̇CO_2_, Table 4). This was expected as repeated human exercise testing is influenced by both technological and biological variability, whereas repeated simulator testing is influenced solely by technological variability. The between‐day variability in V̇O_2_ during human testing is, however, in line with previous work showing variability to range between 1.4% and 8.5% between days [45, 46, 59, 60].

We determined the contribution of technological and biological variability to overall human testing variability by dividing the technological variability (CV) obtained during repeated simulation testing by the total human testing variability (CV). Using this approach, the technological error variability accounted for approximately 40% and 30% of the total variability of V̇O_2_ and V̇CO_2_, respectively, whereas the biological error variability accounted for approximately 60% and 70% of the variability in both V̇O_2_ and V̇CO_2_ (Appendix S1). Similar values were observed for the contribution of technological and biological variability to substrate use and total energy expenditure variability.

Interestingly, these numbers indicate a higher contribution of technological error measurement variability to repeated exercise testing variability than previously determined [45, 46, 47, 61, 62]. Katch and colleagues [45], for example, reported a contribution of technological and biological variability of < 10% and > 90% to V̇O_2_ variability, respectively. Similar numbers were reported by Armstrong and Costill [46]. Because between‐day variability during human exercise testing in the present study was similar to previous reports, the higher contribution of technological variability to total test–retest variability in the present study primarily stems from the higher absolute technological variability as opposed to lower biological variability. The higher technological variability may in turn result from different methods used to determine this variability. For example, the variability of repeated V̇O_2_ measurements during simulation in the present study ranged from 0.5% to 3.2% depending on the device chosen vs. 0.38% reported in Katch et al. [45]. However, they assessed this error only at one flow rate, and they may have used data from just one participant to analyze the variability in gas concentrations. Both aspects will contribute to a lower error vs. the present study by resulting in a smaller range in V̇E and gas concentrations, respectively. Moreover, analyzing gas concentration and flow variability independently—as opposed to combined in the present study—may further underestimate technological variability. Similar reasons may explain why our technological variability is higher than reported by Taylor [47] (where technological variability of a Krogh‐type system comprised < 1% of total test–retest variability) and Armstrong and Costill [46] (technological variability of their computerized system comprised < 10% of total test–retest variability). Given the limitations of prior research in determining this technological variability, and the use of older sensor types and response algorithms, our findings likely provide a more accurate indication of the technological variability and its contribution to human test–retest variability in contemporary systems.

Another aspect that may be hypothesized to contribute to the higher technological variability in the present study compared with prior research is the simulator itself: Although between‐day variability in the simulated V̇O_2_ and V̇CO_2_ is, respectively, < 0.025% and < 0.005% when environmental conditions are identical between days, there is always some variability in the environmental conditions that in turn increases variability in the simulated values. Specifically, the metabolic simulator assumes a certain distribution of atmospheric gases (i.e., N_2_ of 79.05%, O_2_ of 20.9%, and CO_2_ of 0.05%) when adding N_2_ and CO_2_ to the gas mixture. Deviations from the assumed distribution can introduce errors in the simulated gas exchange data such as artificially low or high simulated V̇O_2_ or V̇CO_2_. Differences in the atmospheric gas distribution between days can in turn also generate variability in the simulated—and therefore by CPET systems measured gas exchange data, with the simulation being most sensitive to deviations/differences in O_2_ concentrations (Figure S3). However, because atmospheric gas concentrations were very similar on the two study days, the resulting impact is minimal. For example, based on the simulator manufacturer's equations (see Supplemental file), it can be determined that the O_2_ concentration of 20.93% on Day 1 produced a simulated V̇O_2_ that was on average 0.146% higher, whereas 20.88% O_2_ on Day 2 produced a simulated V̇O_2_ that was 0.093% lower. The net difference between days is therefore (0.146 − (−0.093) =) 0.237% of the average simulated V̇O_2_. which in turn is < 10% of the technological variability for KORR and VO_2_ Master (Table 4). For V̇CO_2_, this difference is expected to be even smaller given the lower sensitivity to assumed CO_2_ concentrations. Overall, the variability in simulated gas exchange data is therefore having only a minimal contribution to the technological between‐day variability.

Overall, while our findings indicate that minimizing biological variability (e.g., by doing multiple repeated tests, and by ensuring standardization of controllable factors [63]) has the greatest potential to reduce overall test–retest variability, they also show that technological variability can contribute substantially to the overall variability of human testing. The use of more reliable CPET systems, therefore, also has the potential to contribute meaningfully to increasing the overall reliability of repeated human measurements. Such an observation is in line with other recent observations suggesting that biological variability may be lower than previously anticipated [22].

### 4.5 Strengths and Limitations

This study has several strengths, but also some limitations. Strengths include the combination of both simulated and real human exercise, the use of a relatively high absolute intensity during the cycling experiments to reflect CPET tests in athletes (average V̇O_2_ and V̇CO_2_ of approximately 2200 and 2000 mL min^−1^, respectively), and the randomized counterbalanced design, repeated over 2 days to better assess accuracy, and to evaluate reliability. Further, another strength is the assessment of technological error across a range of simulated exercise intensities and the assessment of combined errors in V̇O_2_ and V̇CO_2_ rather than gas concentrations and volumes in isolation.

A first limitation is that a metabolic simulator does not fully replicate human exercise because it uses dry gases, operates at a lower temperature, and employs a sinusoidal breathing pattern [18, 53]. However, the findings from the simulator experiments generally matched those seen during actual human testing in the present and previous research [18, 44], thus strengthening its relevance for gaining insight into the actual accuracy of CPET systems without the influence of biological variability. Nevertheless, additional errors may arise during human testing in particular due to water vapor [53]. A second limitation is that we assessed only one device from each manufacturer, and given the possible inter‐device variability (e.g., [44, 64]) the findings should be extrapolated to other devices with caution. Similarly, intra‐device variability over time may occur due to, for example, variations in calibration or sensor degradation [65]. Thirdly, some devices may exhibit drift over time [44, 53], but we did not assess the occurrence of drift using prolonged testing and instead used ~1–3 min time periods during the simulation tests or ~4–6 min periods during human testing. Gas and volume calibrations were however repeated following the human trials to informally assess potential drift. The recalibration yielded similar values, suggesting an absence of drift after these short exercise bouts. Finally, two individuals completed only one cycling test session and were therefore excluded from further analysis of accuracy and reliability, leaving only a relatively small and homogenous sample of four individuals for human testing. Although this is comparable to previous studies assessing accuracy and reliability [44, 45, 46], for this reason, we primarily focused our discussion or accuracy on the results obtained with the simulation experiments and used the human experiments primarily as a verification of the simulation experiments. Further, while more repeated human tests could provide an even more accurate indication of human biological variability, we chose to use two tests to best reflect the most common measurement design in practice and science, and because more tests would reduce the feasibility of the experiments.

### 4.6 Implications

Most implications regarding the validity of CPET systems have been discussed in detail previously [18] and will therefore be discussed only briefly. First, the errors in some of the gas exchange outcomes can impact decision‐making in clinical or sports practice. For example, absolute percentage errors of ~8% for V̇O_2_ (e.g., VO2 Master) could result in a fireman not/falsely meeting a predefined V̇O_2max_ value required to continue professional work [66], in patients not/falsely meeting a predefined V̇O_2max_ value advised to undergo major surgery [1], or correctly/falsely delay medical treatment [2]. Similarly, it may impact performance predictions for athletes [67], talent identification outcomes [68], and diagnosis of overtraining syndrome [69]. Proportionally larger errors for gas exchange outcomes with higher flows may also impact exercise demarcation methods, thereby impacting training advice [18]. Such an effect could particularly impact patient populations that require strict control of exercise intensity (e.g., ischemic heart disease or congestive heart failure), but also athletes who may, as a result, be performing a substantial volume of training at suboptimal intensity. When interpreting the outcomes of substrate use, it is important to note that the same equation was used across all devices in the present study to calculate energy expenditure and substrate utilization from V̇O_2_ and V̇CO_2_. The equation used is considered the most accurate to estimate substrate use during exercise as compared to the ^13^C:^12^C ratio technique [70]. Errors in substrate use and total energy expenditure may therefore be larger when the energy derived from carbohydrates and fats or total energy expenditure is estimated by equations default in the CPET system.

The differences in accuracy between different CPET systems also have implications for the determination of outcomes such as running economy and cycling efficiency. To assess the impact of CPET measurement errors on cycling efficiency, we converted the energy expenditure during human exercise testing (Kcal min^−1^) to metabolic power (Watts) and then divided the average mechanical (cycling ergometer) power (161 W) by the average metabolic power across the two measurement days for all subjects (Table S8). When excluding one highly inconsistent system (Calibre), the results ranged between 17.8% and 20.9%, thus showing CPET inaccuracies can significantly influence estimates of cycling efficiency and thereby also explain at least some of the reported variability in cycling efficiency between different studies.

Our findings also have implications related to repeated exercise testing. In this context, it is essential to first discuss a key consideration related to the quantification of reliability. We used the coefficient of variation (CV) because it is often used as a measure of reliability in the sports science and medicine field [71]. This statistic covers approximately 68% of the (in our case, between‐day) variability. For example, the between‐day technological variability of 1.2% in V̇O_2_ observed for Vyntus CPX means that 68% of the differences between two tests are expected to lie between 1.2%. This means that the variability can be larger for still a modest proportion of tests, and this should be kept in mind when applying these numbers to repeated testing. Moreover, it should be emphasized that technological variability constituted approximately 40%–30% of the human test–retest variability in the present study. Therefore, the variability in repeated human testing will be even larger due to additional biological variability. Given that technological error variability comprises a substantial proportion of overall human testing variability, the use of more reliable CPET systems also has the potential to contribute meaningfully to increasing the overall reliability of repeated human measurements. Similarly, the use of a more reliable system can also reduce the sample size requirements to detect differences between conditions [71]. Finally, we determined the between‐day reliability in the present study as this best reflects situations where a participant is assessed pre‐ and post‐intervention, or when assessing participants on different days. Because between‐day variability is larger than within‐day variability [72], the present study's findings should be applied with caution to within‐day applications.

## 5 Conclusion

Errors for V̇E, BF, V̇O_2_, V̇CO_2_, and RER during simulated exercise differed substantially between systems. A significant variability in accuracy was also observed for substrate utilization, suggesting that substrate utilization derived from indirect calorimetry during exercise should be interpreted with particular caution. The observed errors may impact outcomes derived from CPET measurements such as V̇O_2max_, exercise economy, and thresholds inflection points used for zone demarcation.

Our findings also indicate substantial between‐day technological variability for some devices. This impacts the sensitivity to detect small changes in repeated testing of one individual, and it may also affect the ability to compare between or within (e.g., regression) small groups. Overall, our findings show 40%–30% of the human test–retest variability reflects technological variability, with 60%–70% reflecting biological variability for V̇O_2_ and V̇CO_2_, respectively.

## Author Contributions

B.V.H. conceived the study, collected and analyzed the data, and wrote the first draft of the manuscript; T.S. and B.C.B. contributed to data collection and provided comments and edits. F.M. assisted in data processing. All authors approved the final version.

## Funding

The authors have nothing to report.

## Ethics Statement

Ethical approval was obtained from Maastricht University (nr. 2024‐0257).

## Conflicts of Interest

The authors declare no conflicts of interest.

## Supporting information


**Appendix S1:** Supporting Information.

## Data Availability

All data is available from the OpenScience framework at https://osf.io/b3t8f/overview.
