# Supplementary material for: Technological and Biological Reliability, and Validity of Five Different CPET Systems During Simulated and Human Exercise
Source: Scand J Med Sci Sports. 2026 Jan 24;36(1):e70184. doi: 10.1111/sms.70184 (PMC12831196; doi:10.1111/sms.70184)
Supplement: Supplementary file 1 — Appendix S1: Supporting Information. [file SMS-36-e70184-s001.docx]

**Table S1. Mean ± SD absolute percentage errors (%e) for respiratory parameters, averaged over all simulated steps of both days**

| **System** | **%e V̇E** | **%e BF** | **%e V̇O_2_** | **%e V̇CO_2_** | **%e RER** |
| --- | --- | --- | --- | --- | --- |
| Vyntus CPX | 1.20 ± 0.53** | 0.92 ± 0.45** | 3.26 ± 1.55 | 4.07 ± 1.08 | 1.21 ± 1.42* |
| Oxycon Pro | 0.69 ± 0.79** | 0.82 ± 0.46** | 3.12 ± 1.16 | 3.15 ± 0.44 | 1.37 ± 1.69 |
| KORR | 5.65 ± 3.43 | 1.11 ± 0.60** | 6.03 ± 2.14 | 12.1 ± 5.37 | 6.94 ± 4.87 |
| VO2masterPro | 0.93 ± 0.45** | 1.44 ± 0.70* | 7.86 ± 4.53 | n.a. | n.a. |

BF = breathing frequency; RER = respiratory exchange ratio; VE = ventilation; V̇CO_2_ = rate of carbon dioxide production; V̇O_2_ = rate of oxygen consumption.

** good agreement (<3% error); * acceptable agreement (<5% error); No star indicates that we were unable to establish good or acceptable agreement.

**Table S2. Mean ± standard deviation absolute percentage errors (%e) for substrate use and total energy expenditure, averaged over all simulated steps of both days**

| **System** | **%e Energy from carbs** | **%e Energy from fats** | **%e Total energy expenditure** |
| --- | --- | --- | --- |
| Vyntus CPX | 2.83 ± 4.20 | 14.1 ± 13.4 | 4.21 ± 0.71* |
| Oxycon Pro | 8.19 ± 9.38 | 15.3 ± 15.2 | 4.46 ± 0.32* |
| KORR | 48.8 ± 26.4 | 50.3 ± 43.8 | 6.98 ± 2.44 |
| VO2masterPro | n.a. | n.a. | n.a. |

** good agreement (<5% error); * acceptable agreement (<10% error); No star indicates that we were unable to establish good or acceptable agreement.


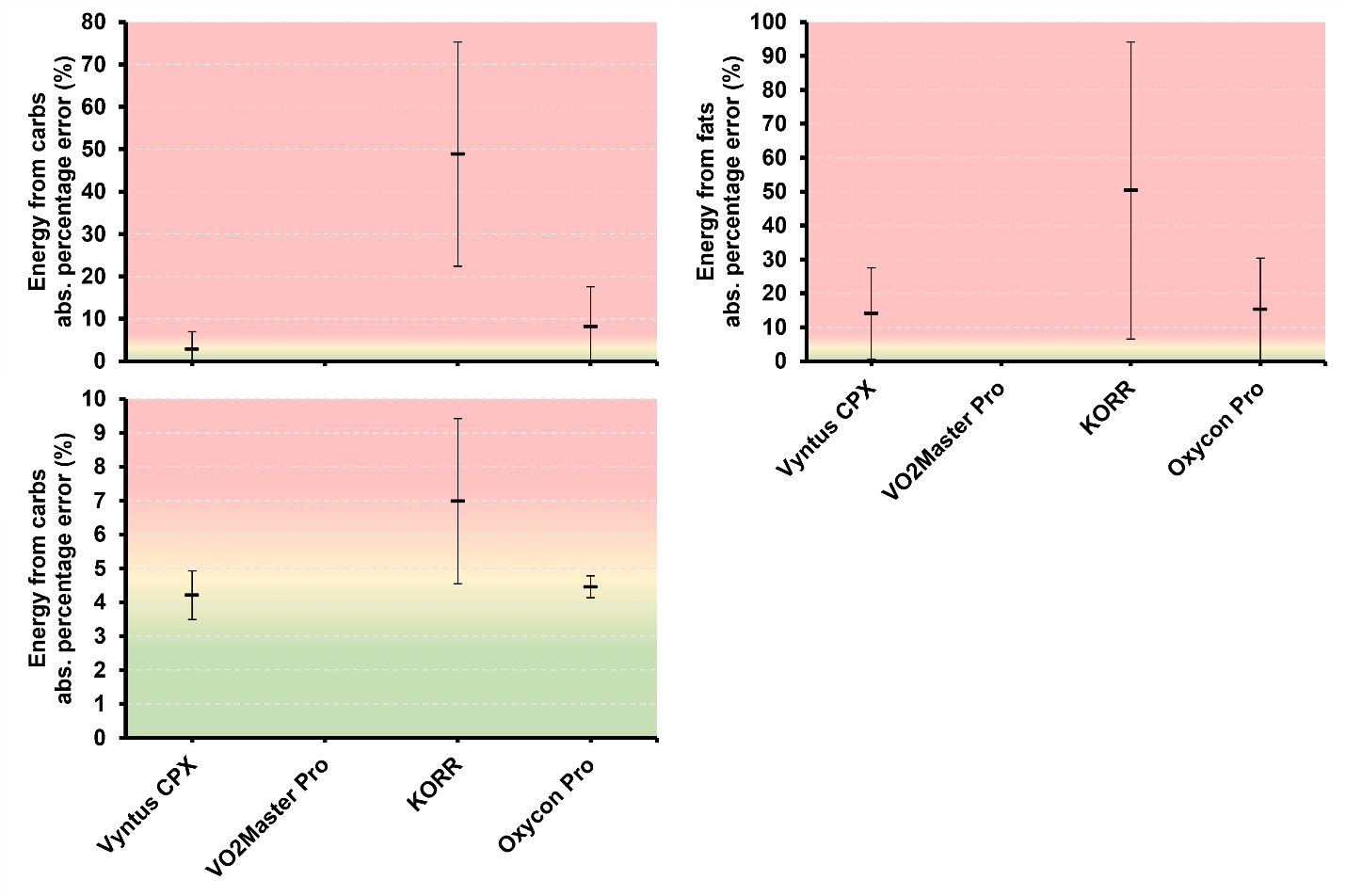


**Figure S1. Absolute percentage errors for energy derived from carbohydrates, energy derived from fats, and total energy expenditure. Horizontal lines represent the average error over all simulated steps over both days, while error bars represent the standard deviation of the error over all simulated steps of both days. Wider error bars indicate a lower precision of the measured variable. No error for substrate usage or total energy expenditure is available for VO2masterPro as this device measures only V̇O_2_.**

**Table S3. Outcome of the statistical test which assesses whether the slope of the linear regression of the relative percentage error for** **V̇O_2_ and V̇CO_2_, differs from zero**

| **System** | ***p*-value for slope of V̇O_2_ error** | **Magnitude (change in %e per mL•min^-1^ increase in VO_2_); direction slope** | ***p*-value for slope of V̇CO_2_ error** | **Magnitude (change in %e per mL•min^-1^ increase in VCO_2_); direction slope** |
| --- | --- | --- | --- | --- |
| Vyntus CPX | 0.02* | -0.001, ↓ | 0.70 | <0.001, ↔ |
| Oxycon Pro | 0.13 | -0.001, ↔ | 0.90 | <0.001, ↔ |
| KORR | 0.90 | <0.001, ↔ | 0.70 | <0.001, ↔ |
| VO2masterPro | 0.62 | <0.001, ↔ | n.a. | n.a. |

* indicates significant differences *p* < .05

↓ and ↑ represent a significant decrease and increase in the measured V̇O_2_ and V̇CO_2_ with higher simulated V̇O_2_ and V̇CO_2_, respectively, while ↔ represents no change. Note that decreases or increases can indicate that the error decreases depending on the error at the lower simulated value. For example, the error for V̇O_2_ for Vyntus CPX decreases with higher volumes due to the negative slope.
Abbreviations; V̇CO_2_ = rate of carbon dioxide production; V̇O_2_ = rate of oxygen uptake.

**Table S4. Mean ± SD heart rate during the human experiments for day one and day two per system**

| **System** | **Heart rate (beats∙min^-1^) day one** | **Heart rate (beats∙min^-1^) day two** |
| --- | --- | --- |
| Vyntus CPX | 119 ± 9.63 | 119 ± 13.2 |
| Oxycon Pro | 117 ± 9.50 | 117 ± 10.9 |
| VO2master Pro | 117 ± 9.57 | 119 ± 11.8 |
| Calibre | 117 ± 9.69 | 117 ± 10.5 |
| KORR | 118 ± 9.32 | 118 ± 10.4 |

**Table S5. Mean V̇O_2_, V̇CO_2_, substrate use, and total energy expenditure measured during the two randomized and counterbalanced cycling experiments for all systems (averaged over both days)**

| **System** | **V̇O_2_ (mL∙min^-1^)** | **V̇CO_2_ (mL∙min^-1^)** | **V̇O_2_ (% relative to reference value)** | **V̇CO_2_ (% relative to reference value)** | **Energy from carbs (Kcal∙min^-1^)** | **Energy from fats (Kcal∙min^-1^)** | **Total energy expenditure (Kcal∙min^-1^)** | |
| --- | --- | --- | --- | --- | --- | --- | --- | --- |
| Vyntus CPX | 2372 ± 425 | 2070 ± 440 | 6.87 | 1.71 | 4.91 ± 2.58 | 5.26 ± 1.08 | 10.2 ± 2.14 |  |
| Oxycon Pro | 2204 ± 397 | 2153 ± 479 | -0.68 | 5.76 | 7.90 ± 3.54 | 1.83 ± 1.72 | 9.73 ± 2.03 |  |
| VO2master Pro | 2405 ± 533 |  | 8.37 |  |  |  |  |  |
| Calibre | 1329 ± 742 | 1232 ± 846 | -40.1 | -39.5 | 2.50 ± 5.58 | 2.74 ± 1.90 | 5.24 ± 3.78 |  |
| KORR | 2604 ± 506 | 2419 ± 573 | 17.3 | 18.9 | 7.35 ± 3.83 | 3.91 ± 1.56 | 11.3 ± 2.58 |  |

**Table S6. Between-day variability in original and percentage units from the human cycling experiments**

| **System** | **V̇E (L∙min^-1^)** | **V̇O_2_ (mL∙min^-1^)** | **V̇CO_2_ (mL∙min^-1^)** | **RER** | **Energy from carbs (Kcal∙min^-1^)** | **Energy from fats (Kcal∙min^-1^)** | **Total energy expenditure (Kcal∙min^-1^)** |
| --- | --- | --- | --- | --- | --- | --- | --- |
| **Original units** |  |  |  |  |  |  |  |
| Vyntus CPX | 2.28 | 74.8 | 102 | 0.06 | 2.11 | 2.27 | 0.33 |
| Oxycon Pro MC | 5.90 | 271 | 135 | 0.08 | 1.85 | 2.90 | 1.28 |
| KORR | 3.25 | 67.4 | 156 | 0.04 | 2.13 | 1.90 | 0.37 |
| VO2masterPro | 0.60 | 181 |  |  |  |  |  |
| Calibre | 16.3 | 1036 | 983 | 0.08 | 5.42 | 3.78 | 5.16 |
|  | **V̇E (%)** | **V̇O_2_ (%)** | **V̇CO_2_ (%)** | **RER** | **Energy from carbs (%)** | **Energy from fats (%)** | **Total energy expenditure (%)** |
| **Percentage units (CV)** |  |  |  |  |  |  |  |
| Vyntus CPX | 4.14 | 3.15 | 4.95 | 6.34 | 30.7 | 46.6 | 2.80 |
| Oxycon Pro MC | 11.2 | 12.3 | 6.27 | 8.31 | 18.0 | 28.1 | 12.4 |
| KORR | 5.61 | 2.59 | 6.46 | 4.80 | 21.2 | 65.0 | 2.86 |
| VO2masterPro | 1.06 | 7.54 |  |  |  |  |  |
| Calibre | 29.7 | 77.9 | 79.8 | 10.2 | 107 | 247 | 78.0 |

Abbreviations: MC = mixing chamber; RER = respiratory exchange ratio; V̇E = ventilation; V̇CO_2_ = rate of carbon dioxide production; V̇O_2_ = rate of oxygen uptake.


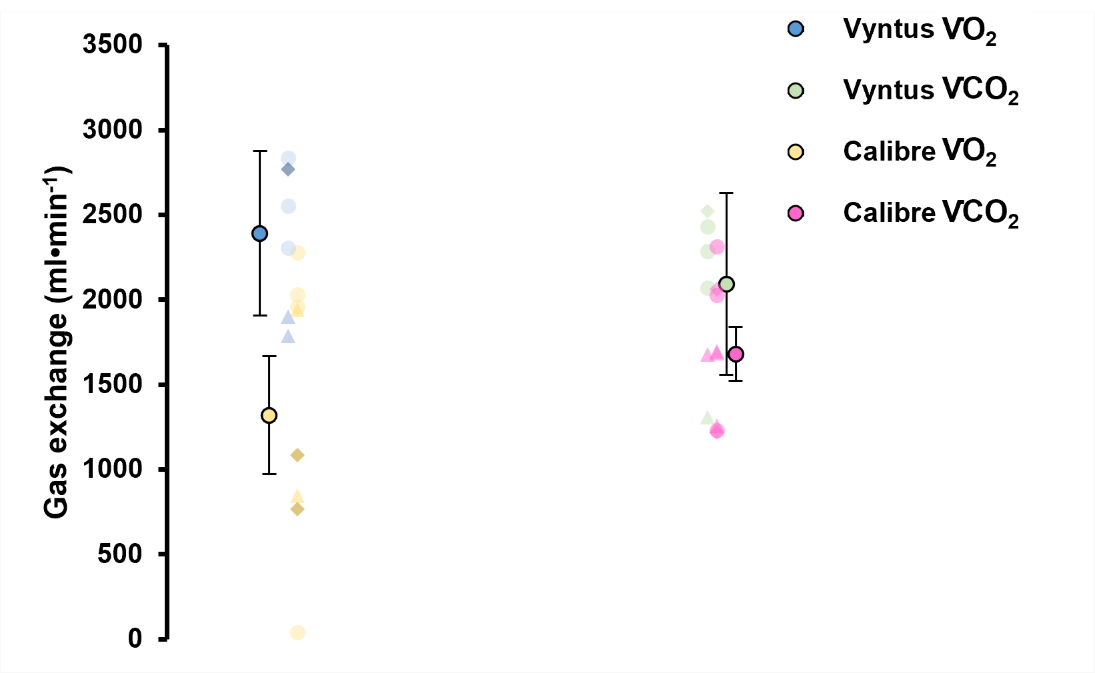


**Figure S2. Gas exchange data during the additional repeated-measures testing over 2 days for Calibre and Vyntus CPX in three well-trained participants. Dots depict the mean value with error bars the standard deviation across the participants. Symbols show individual datapoints, with a color and shape assigned to each participant. For example, the blue and yellow circles depict the V̇O2 for Vyntus CPX and Calibre for participant 1, respectively.**

**Table S7. Gas exchange data for the first and second Calibre re-test**

|  | **V̇E (L∙min^-1^)** | **V̇O_2_ (mL∙min^-1^)** | **V̇CO_2_ (mL∙min^-1^)** |
| --- | --- | --- | --- |
| **1^st^ re-test** |  |  |  |
| Vyntus CPX | 58.4 ± 15.5 | 2391 ± 485 | 2092 ± 536 |
| Calibre | 55.2 ± 9.72 | 1320 ± 346 | 1681 ± 159 |
| **2^nd^re-test** |  |  |  |
| Vyntus CPX | 60.9 ± 11.4 | 2369 ± 563 | 2107 ± 570 |
| Calibre (10 min continuous)* | 61.0 ± 15.9 | 2009 ± 477 | 2062 ± 568 |
| Calibre (2×5 min)* | 64.5 ± 16.2 | 2164 ± 451 | 2211 ± 597 |

* Note that on average the gas exchange data for the second test looks relatively similar, but this is because it was both underestimated and overestimated at the individual level, masking group-level differences.

Full data for the 2^nd^ re-test is available from [**https://doi.org/10.17605/OSF.IO/B3T8F**](https://doi.org/10.17605/OSF.IO/B3T8F)

**Table S8. Metabolic power, cycling mechanical power, and cycling efficiency across the different CPET systems**

| **System** | **Mean metabolic power (Watts)** | **Mean cycling power (Watts)** | **Mean cycling efficiency (%)** |
| --- | --- | --- | --- |
| Vyntus CPX | 819 | 161 | 19.7 |
| Oxycon Pro MC | 770 | 161 | 20.9 |
| KORR | 905 | 161 | 17.8 |
| VO2 Master Pro | - | 161 | - |
| Calibre | 461 | 161 | 34.9 |

The metabolic simulator assumes fixed ambient gas fractions ($\hat{p}_{N_{2}}=0.7905$, $\hat{p}_{O_{2}}=0.2090$, $\hat{p}_{CO_{2}}=0.0005$, $\hat{p}_{rest}=0.0000$) when adding N_2_ and CO_2_ to the gas. Deviations between these assumed and the true ambient concentrations introduce systematic errors in the simulated gas exchange. Although resulting errors in VCO₂ are generally small under typical conditions, deviations in ambient oxygen concentration lead to larger errors in VO₂ (Figure S3). This difference in sensitivity to O_2_ and CO_2_ concentrations arises because the absolute amount of CO₂ injected by the simulator is large relative to the ambient CO₂ concentration, rendering small variations in environmental CO₂ negligible relative to O_2_. The relative deviations introduced by mismatches between assumed and actual gas fractions can be determined using the equations below:

$$\Delta{VO2}_{set-simulated}\left[ \% \right]=-\frac{\hat{p}_{O2}-p_{O2}}{\hat{p}_{O2}}$$

$$\Delta{VCO2}_{set-simulated}\left[ \% \right]=\frac{\hat{p}_{CO2}-p_{CO2}}{RER*\hat{p}_{O2}}$$

Where $\hat{p}_{O2}$ represents the assumed ambient oxygen fraction, $\hat{p}_{CO2}$ the assumed ambient carbon dioxide fraction, and $p_{O2}$ and $p_{CO2}$ represent the actual fractions.


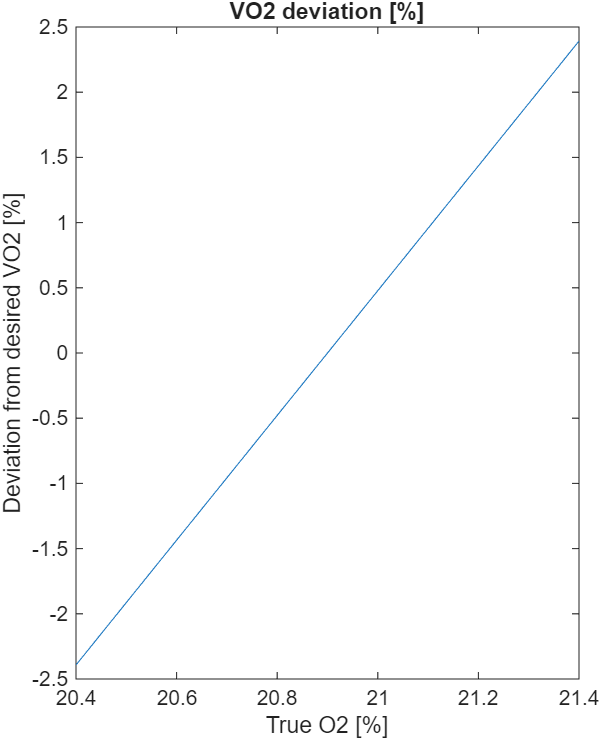

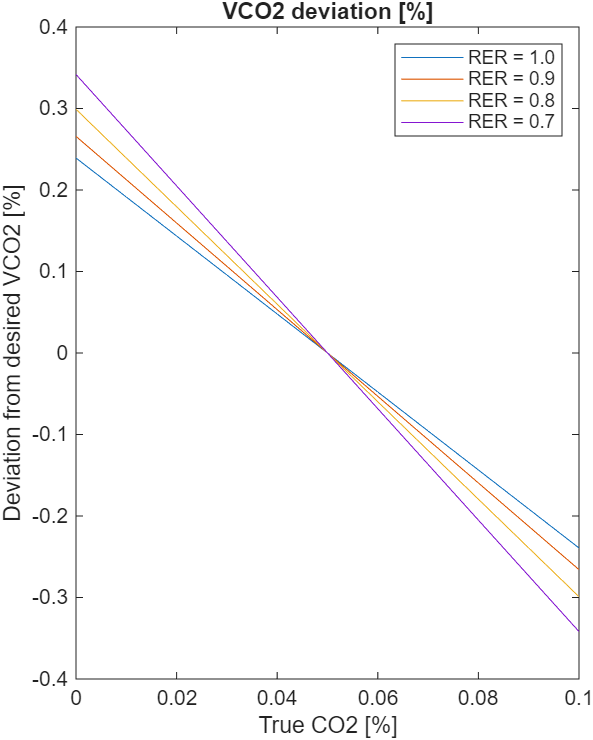


**Figure S3. Errors introduced in the simulated V̇O_2_ and V̇CO_2_ by deviations in ambient gas concentrations from assumed gas concentrations.**
